# Successful transcatheter occlusion of an aortopulmonary window using a PFM coil in a pediatric patient: a case report and literature review

**DOI:** 10.1186/s13019-025-03735-w

**Published:** 2025-12-29

**Authors:** Hojjat Mortezaeian, Maryam Taheri, Mohammadreza Taheri, Mohsen Anafje

**Affiliations:** 1 Interventional Research Center, Rajaei Cardiovascular, Medical and Research Institute, IUMS, Tehran, Iran; 2 Cardiovascular Disease Research Center, School of Medicine, Hamadan University of Medical Sciences, Hamadan, Iran; 3 Cardiovascular Research Center, School of Medicine, Iran University of Medical Sciences, Tehran, Iran; 4 Rajaie Cardiovascular Medical and Research Institute, School of Medicine, Iran University of Medical Sciences, Tehran, Iran

**Keywords:** Aortopulmonary window, Congenital heart defects, Pulmonary hypertension, Cardiac catheterization, Transcatheter occlusion, Case report

## Abstract

**Introduction:**

Aortopulmonary window (APW) is a rare congenital heart defect that causes left-to-right shunting and pulmonary overcirculation. Appropriate intervention prevents long-term complications.

**Case presentation:**

We report a 3-year-old Iranian girl (weight ~ 14 kg, height ~ 95 cm) presenting with exertional dyspnea, systolic murmur, and bounding pulses. Echocardiography showed a 3 mm Type I APW with mild pulmonary hypertension. Pre-procedure catheterization revealed a mean pulmonary artery pressure (PAP) of 26 mmHg and a Qp/Qs ratio of 1.8. The defect was closed percutaneously using a 5 × 4 mm PFM coil. No pre-procedure CT scan was performed. Post-procedural evaluation demonstrated complete closure, mean PAP reduced to 18 mmHg, Qp/Qs normalized to 1.0, and no residual shunt.

**Conclusion:**

Transcatheter coil occlusion can be a safe and effective option for small APWs, offering favorable outcomes without the risks of open-heart surgery.

## Introduction

Aortopulmonary window (APW) is a rare congenital defect, accounting for ~ 0.2% of congenital heart disease cases in children [1]. APW involves abnormal communication between the ascending aorta and the pulmonary artery, which leads to a significant left-to-right shunt [2, 3]. APW is classified into three types: Type I, a proximal defect between the ascending aorta and main pulmonary artery (the most common form); Type II, a distal defect near the origin of the right pulmonary artery; and Type III, a large defect combining features of both. Our patient had a Type I APW [4]. While many APW cases occur in isolation, up to half are associated with other defects, such as atrial septal defect (ASD), ventricular septal defect (VSD), interrupted aortic arch (IAA), coarctation of the aorta, and tetralogy of Fallot (TOF) [5]. In severe cases of untreated APW, irreversible pulmonary hypertension and heart failure can significantly worsen the prognosis. Surgical repair remains the standard treatment, particularly in early diagnosed cases [6], although device-based transcatheter approaches have been reported in selected patients [7]. Here, we describe a pediatric case of successful transcatheter coil occlusion of a small APW, highlighting its feasibility as a minimally invasive alternative to surgery.

## Case presentation

A previously healthy 3-year-old Iranian girl (~ 14 kg, ~ 95 cm) was referred after a systolic murmur was detected during routine examination. Her parents reported mild exertional dyspnea but normal growth and development, with no history of cyanosis, palpitations, syncope, or congenital heart disease.

On examination, she appeared well nourished and in no distress. Vital signs were age-appropriate: Heart rate (HR) 98/min, Respiratory rate (RR) 22/min, Blood pressure (BP) 95/60 mmHg, Temperature 37.2 °C, and Oxygen Saturation (SpO₂) 98% on room air. Cardiovascular exam revealed a normal S1, a loud P2, and a grade III/VI systolic ejection murmur at the left sternal border. Lungs were clear, and there were no signs of retractions, cyanosis, or clubbing. The abdominal, neurologic, and extremity exams were unremarkable.

### Diagnostic workshop and interventional procedure

On initial exam, a murmur suggesting a left-to-right shunt prompted transthoracic echocardiography, which revealed a ~ 3 mm Type I APW with mild pulmonary hypertension, mild LV dilation, and preserved systolic function (Ejection fraction ~ 70%). End-diastolic forward flow and pan-diastolic antegrade flow were present (Table 1). Pre-procedural CT angiography confirmed a small proximal APW without coronary anomalies, supporting candidacy for transcatheter closure.

The patient underwent right and left heart catheterization via right femoral venous and arterial access. Angiography confirmed a ~ 3 mm Type I APW with direct aortopulmonary communication, elevated pulmonary artery pressure, and a significant left-to-right shunt (Qp: Qs 1.8:1) (Table 1). Aortic root injections in LAO and AP views delineated the defect and its safe distance from the left main coronary artery.

**Fig. 1:**
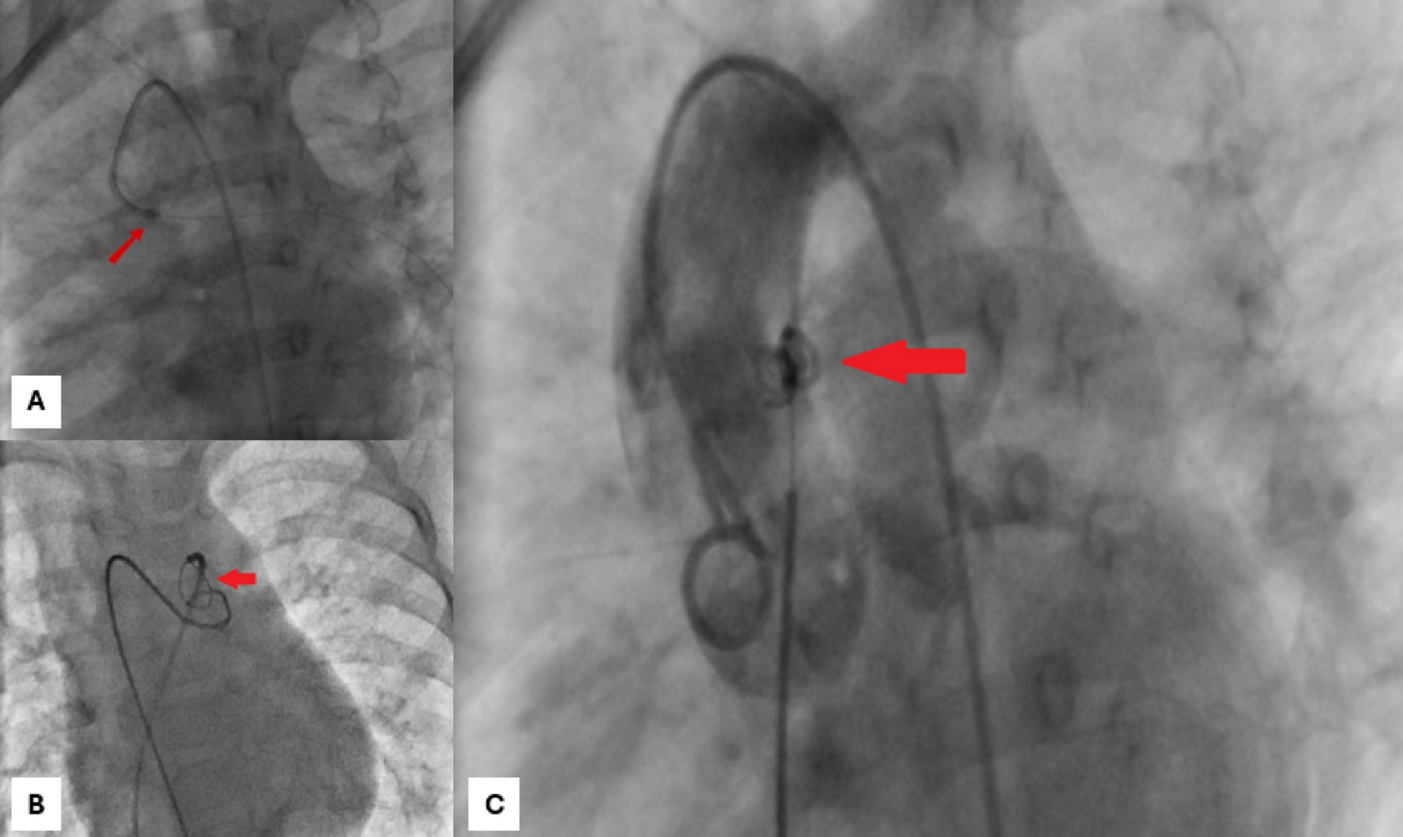
Transcatheter Coil Occlusion of the Aortopulmonary Window (APW). (**A**) Fluoroscopic image showing a guidewire (red arrow) introduced via the femoral artery, traversing the APW into the pulmonary artery. (**B**) Creation of an arteriovenous loop: the wire is snared from the venous side to stabilize access across the defect. (**C**) Final coil placement (red arrow) across the APW, demonstrating precise positioning, complete occlusion of the defect, and preserved flow in adjacent structures

The case was reviewed in a multidisciplinary team meeting, and after discussion, percutaneous closure was recommended. Written informed consent was obtained from the parents. Under fluoroscopic guidance, a 4 Fr JR catheter and arteriovenous loop facilitated coil delivery. A 5 × 4 mm PFM coil was deployed across the APW, achieving immediate occlusion without compromising adjacent structures (Figs.1A-C).

**Fig. 2:**
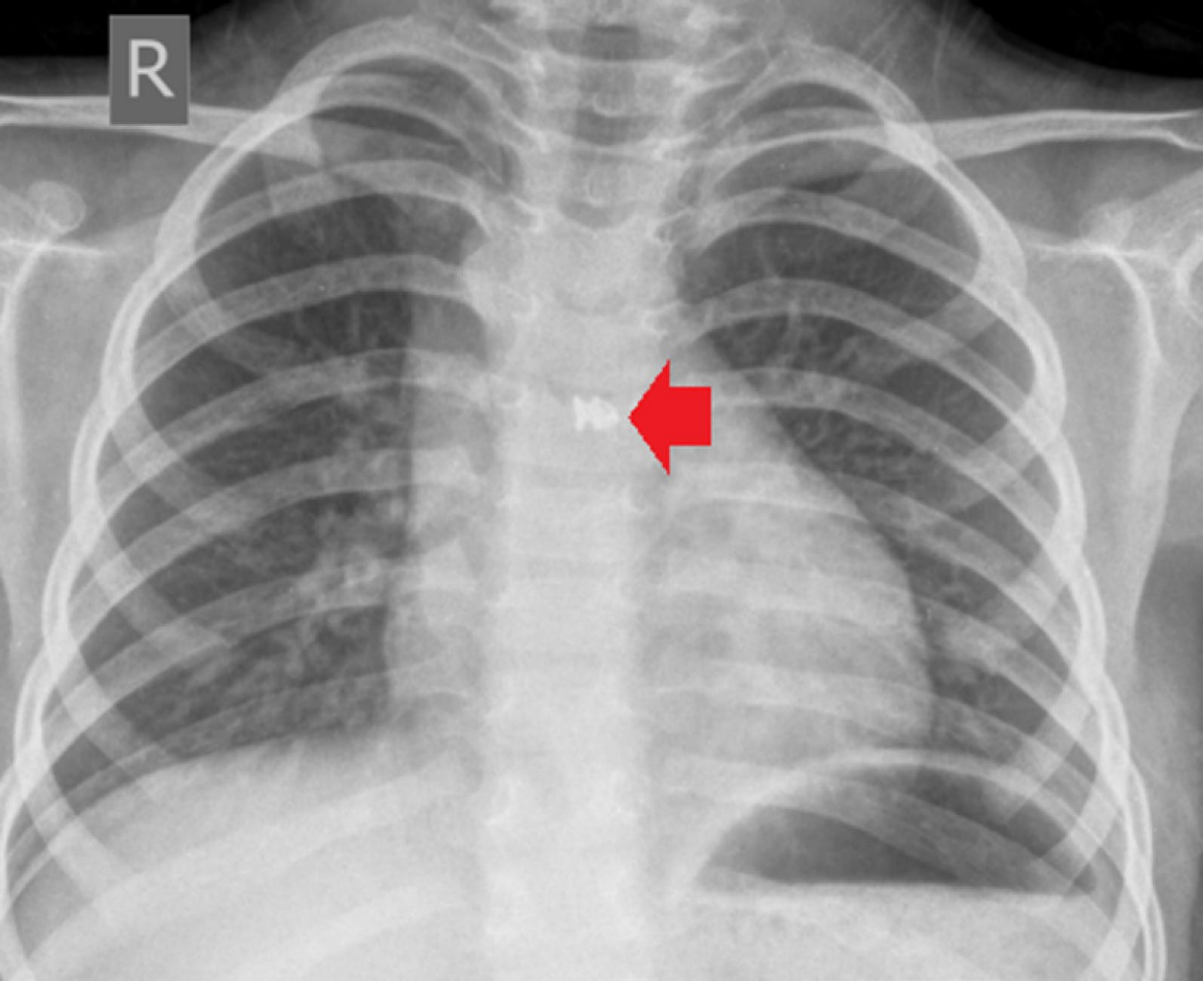
Post-procedure Chest X-ray (CXR): The post-procedure CXR shows the successful deployment of a coil (indicated by the red arrow) used to close the aortopulmonary window (APW). The coil is well-positioned in the area of the former communication between the ascending aorta and pulmonary artery

Post-deployment angiography and chest radiography confirmed stable positioning and absence of residual shunt (Fig. 2). Echocardiography showed complete closure, preserved aortic and pulmonary valve function, and normal LV systolic function (EF ~ 70%). Repeat hemodynamic assessment demonstrated no step-up and reduced pulmonary pressures, consistent with effective defect closure (Table 1).

## Results and follow-up

Diagnostic and interventional catheterization achieved definitive closure of the APW without surgery. The patient recovered uneventfully and maintained normal growth and activity.

At 6 months, echocardiography confirmed complete closure, normalization of pulmonary pressures, and improved chamber dimensions. Chest radiography showed reduced pulmonary congestion and no cardiomegaly. Ongoing surveillance will assess residual shunt, pulmonary vascular disease, coronary compromise, or coil migration, though none are expected to have a stable early outcome.

## **Discussion**

The present case demonstrates that transcatheter coil occlusion can achieve complete closure of a moderate APW in a child, underscoring its value as a minimally invasive alternative to surgery in carefully selected patients.

Echocardiography with color Doppler remains the gold standard for diagnosing APW, allowing direct visualization of the defect, assessment of shunt magnitude, and detection of associated anomalies. Angiography further defines anatomy in complex cases and is essential for planning transcatheter or surgical repair [8].

While surgical repair remains the standard treatment for APW [6], advances in occluder technology have made transcatheter closure a viable alternative in carefully selected patients. Favorable anatomy—typically small, type I defects with adequate rims and safe distance from coronary and valvular structures—allows successful percutaneous closure, offering a less invasive option and shorter recovery compared with surgery [9]. Coil embolization, as used in our case, is an effective option for small to moderate APWs, offering a minimally invasive alternative that reduces pulmonary overcirculation and avoids the risks of open-heart surgery [10].

Other transcatheter techniques, such as Gore-Tex patch ‘sandwich’ closure between the aorta and pulmonary artery, provide a less invasive alternative to surgery and have shown success in complex cases while reducing intraoperative risk [11]. The Amplatzer™ device has been widely used in congenital heart disease [12] and effective for APW closure, particularly in infants with suitable anatomy, and surgical techniques also provide durable outcomes [13].

In our case, the small, tubular defect was better suited to coil occlusion. A PFM coil offered secure anchoring, minimized risk to adjacent structures, and provided a simpler, less invasive solution than larger occluders, achieving complete closure with an excellent result. Transcatheter closure carries risks such as device embolization, great vessel obstruction, and valvular interference, underscoring the importance of careful patient selection [14].

APW produces a left-to-right shunt that increases pulmonary blood flow and pressure, leading to pulmonary hypertension and, if untreated, eventual Eisenmenger physiology with irreversible vascular damage and poor prognosis [2]. Even in cases complicated by severe pulmonary arterial hypertension, surgical correction has demonstrated promising results, significantly improving outcomes and optimizing survival while preventing life-threatening complications [6]. Previous reports highlight the spectrum of APW outcomes, from fatal progression with delayed diagnosis [15] to favorable results after timely repair [16]. These cases reinforce the importance of early detection and individualized management to prevent irreversible complications.

Table 2 summarizes reported APW cases, underscoring the variability in presentation and management.

**Table 1:** Comparison of Pre- and Post-procedure echocardiography and angiography findings

Parameter	Pre-procedure echocardiography	Post-procedure echocardiography
APW	Moderate-sized (3 mm) APW, Type 1	Complete closure, no residual shunt
Mean PAP (by Echo*)	26 mmHg (estimated by TR jet, RV-RA gradient)	18 mmHg (Same method)
LVEF/LV dimensions	70%; LVEDD 52 mm (mildly dilated, Z + 2.1)	70%; LVEDD 48 mm (Z + 1.5, improved)
Aortic/Pulmonary valve function	Normal	Normal
Pulmonary hypertension	Present, mild	Resolved, no evidence of pulmonary hypertension
EDF and pan diastolic anterograde flow	Present	Absent

Parameter	Pre-procedure angiography	Post-procedure angiography
APW	Moderate-sized (3 mm) APW, Type 1, significant left-to-right shunt	Coil occluder deployed, no residual shunt
PAP	26 mmHg	18 mmHg mean pressure
Qp/Qs*	1.8:1	1.0
Shunt direction	Left-to-right	No shunt
Coil placement	N/A	Coil successfully placed in the APW
Coronary arteries	No abnormalities noted	No abnormalities noted

APW: Aortopulmonary Window, EDF: End-diastolic forward flow, LV: Left Ventricle, LVEDD: Left Ventricular End Diastolic Diameter, LVEF: Left Ventricular EF, N/A: Not Applicable, PAP: Pulmonary Artery Pressure, RA: Right Atrium, RV: Right Ventricle, Qp/Qs: Pulmonary-systemic shunt ratio, TR: Tricuspid regurgitation

*Mean PAP estimated non-invasively from tricuspid regurgitation velocity and pulmonary flow Doppler indices

Qp/Qs was calculated using the Fick principle based on oxygen saturation data obtained during cardiac catheterization (step-up between RV, PA, and systemic arterial saturations)

**Table 2:** Summary of case reports involving aortopulmonary window (APW) management

Authors & YOP	Gender/ Age	CC, HX, PH/E	Imaging	Dx	TX, Prog
Chavan et al. 2024 [17]	F/3 years	CC: Heart murmur on routine exam. HX: Previously healthy; mild exertional dyspnea, no cyanosis or syncope. PH/E: Well-nourished, stable vitals; loud P2, grade III/VI systolic ejection murmur, no cyanosis or clubbing.	Echo: 3 mm Type I APW, mild pulmonary HTN, mild LV dilation, EF 70%. CT: Small proximal APW, normal coronaries. Cath: Qp/Qs 1.8:1, PAP mildly elevated.	Small, Type I APW with left-to-right shunt.	Tx: Transcatheter coil closure with 5×4 mm PFM coil. Complete occlusion, no residual shunt. Prog: At 6-month follow-up, normal growth, resolution of pulmonary HTN, no complications.
Ten et al., 2023 [5]	M/Newborn	CC: Heart murmur, respiratory distress. HX: Prematurity (32 weeks), diagnosed with APW type I and IAA type A. PH/E: Oliguria, weak femoral pulses post-op.	Echo: APW type I, IAA. Post-op arch obstruction.	APW type I, IAA type A.	TX: Single-stage repair of APW and IAA with autologous pericardial patches, re-intervention on POD 1 due to arch obstruction. Prog: Asymptomatic at 6-year follow-up, mild arch stenosis.
Ali et al., 2022 [18]	F/12 months	CC: Failure to thrive. HX: Congenital heart defect. PH/E: Weight 7 kg, hyperdynamic heart, machinery murmur grade 5.	Echo: APW (5 mm), LV/LA dilated, severe pulm. HTN (PAP 50 mmHg).	Large APW with severe pulmonary hypertension	TX: Transcatheter closure with Multifunctional Occluder (Konar). Prog: No residual flow, discharged after 24 hours.
Guzeltas et al., 2021 [13]	M/1 month, F/6 months	CC: Tachypnea, dysphagia, murmur in both cases. HX: Diagnosed with APW.	Echo: APW (Type IV), CT revealed tubular APW.	APW (Type IV).	TX: Transcatheter closure using Amplatzer Duct Occluder-I (ADO-I) device. Prog: No residual defects, successful closure, stable condition.
Giordano & Butera, 2020 [7]	F/25	CC: Dyspnea on exertion during pregnancy.PH/E: Systolic murmur, systemic arterial pressure 120/50 mmHg, LV dilation (69 mm), mild systolic dysfunction (EF 45%).	Echo: APW (9 mm), L-to-R shunt, PAP 40 mmHg.	Large APW	TX: Transcatheter closure using Amplatzer Muscular Septal Occluder (16 mm) under fluoroscopy and balloon sizing. Prog: Asymptomatic, device well-positioned at 1-year follow-up, LV function normalized.

ADO-I: Amplatzer Duct Occluder-I, APW: Aortopulmonary Window, ASD: Atrial Septal Defect, CC: Chief Complaint, CT: Computed Tomography, Dx: Diagnosis, EF: Ejection Fraction, F: Female, HTN: Hypertension, Hx: History, IAA: Interrupted Aortic Arch, L: Left, LA: Left Atrium, LV: Left Ventricle, M: Male, PAP: Pulmonary Artery Pressure, PH/E: Physical Examination, POD: Postoperative Day, Prog: Prognosis, R: Right, TX: Treatment, YOP: Year of Publication

## Conclusion

Transcatheter coil occlusion is a safe and effective minimally invasive option for selected cases of APW with suitable anatomy. This approach achieves complete closure while avoiding the risks of open-heart surgery, highlighting its role as a valuable alternative to surgical repair.

## Data Availability

No datasets were generated or analysed during the current study.
